# Supplementary material for: Global soil antibiotic resistance genes are associated with increasing risk and connectivity to human resistome
Source: Nat Commun. 2025 Aug 4;16:7141. doi: 10.1038/s41467-025-61606-3 (PMC12322111; doi:10.1038/s41467-025-61606-3)
Supplement: Supplementary file 1 — Supplementary Information [file 41467_2025_61606_MOESM1_ESM.pdf]

Global soil antibiotic resistance genes are associated with increasing risk and connectivity to human resistome

Yuxiang Zhao[a], Liguan Li[a], Yue Huang[a], Xiaoqing Xu[a], Zishu Liu[b], Shuxian Li[a], Lizhong Zhu[b], Baolan Hu[b, c *], Tong Zhang[a, d, e, f, g *]

[a] Environmental Microbiome Engineering and Biotechnology Laboratory, Department of Civil Engineering, The University of Hong Kong, Hong Kong SAR, China

[b] College of Environmental and Resource Sciences, Zhejiang University, Hangzhou, China

[c] Zhejiang Province Key Laboratory for Water Pollution Control and Environmental Safety, Hangzhou, China

[d] School of Public Health, The University of Hong Kong, Hong Kong SAR, China

[e] Center for Environmental Engineering Research, The University of Hong Kong, Hong Kong SAR, China

[f] Shenzhen Innovation and Research Institute, The University of Hong Kong, Shenzhen, China

[g] The State Key Laboratory of Marine Environmental Health (SKLMEH), City University of Hong Kong, Hong Kong SAR, China

[#]For correspondence

Tong Zhang

E-mail: zhangt@hku.cn

Baolan Hu

E-mail: blhu@zju.edu.cn

Text S1 Rarefaction analysis

We conducted rarefaction analysis for the different habitats (Fig. S3) to determine whether the used samples were representative (reaching a plateau) and the diversity across various habitats. The rarefaction analysis was performed using the R packages "*vegan*", "*doBy*", "*knitr*", "*dplyr*", "*purrr*" and "*ggalt*". To ensure the reliability of the results, we analyzed all levels, including type, subtype, and RankI ARG genes.

Text S2 RankI ARGs

ARGs are widespread among bacteria. However, not all ARGs pose a serious threat to public health, highlighting the importance of identifying those that are high-risk[1]. An 'omics-based' framework was established to evaluate ARG risk considering human-associated-enrichment, gene mobility, and host pathogenicity. This last version including 225 RankI ARGs genes, and the ARGs related to multidrug were excluded. Overall, 214 RankI ARGs genes were included in the analysis (Supplementary Data 3). 36 (total of 37) Rank I ARG families highlighted by World Health Organization (WHO) were included in this list. Only *vanA* were excluded, due to their universality and abundance in "non-human-associated" environments.

Text S3 Adonis analysis of ARGs

We used Adonis analysis to test the differences of ARG composition between various habitats. We tested the difference of gene type. The Adonis analysis was carried out on the basis of Bray-Curtis distance using the R package "*vegan*"[2]. As we focused on both total ARGs and RankI ARGs, each was analyzed. Results showed

that significant differences could be observed in all tested groups (Table S1).

The significance in Adonis analysis might be caused by homogeneous dispersion or heterogeneous dispersion. Only homogeneous dispersion meets the assumption for Adonis analysis, therefore, we further calculated multivariate homogeneity of group dispersions (variances) to test the homogeneity of dispersion among groups using the R package "*vegan*". No significance (p > 0.05) was observed at any of the levels tested, indicating that the result was influenced by the difference in composition between groups (homogeneous dispersion) rather than within groups (heterogeneous dispersion) (Table S2). Thus, the significant differences obtained by Adonis analysis met its assumption, and were reliable. Therefore, there were significant differences in resistance genes between habitats, both for all ARGs and RankI ARGs (Supplementary Data 4 - Supplementary Data 5).

Text S4 Biomaker ARGs in soil

We further used LEfSe analysis to reveal the biomaker ARGs in different habitats, using "*pacman*", "*tidyverse*", "*microecom*", and "*agrittr*". Results showed that soil possessed biomaker ARGs at both the type and subtype levels. But, no biomaker RankI genes were observed in soil (Supplementary Data 6).

Text S5 Determining how fungal reads influence ARGs-OAP analyses

Theoretically, ARGs-OAP focuses only on bacterial ARGs, however as fungi are one of the key organismal groups in the soil, it is necessary to determine the effect of fungal reads on ARGs-OAP analysis. We selected a subset of data (15 metagenomic samples), including 5 land use types (grassland, forest, farmland, construction land, and bare land), to test the effect of soil fungi on ARGs-OAP. After quality control, we used kraken2 to identify fungi at the read level and extract the corresponding sequences. We used the fungal sequences to construct a fungal dataset, and further analyzed the ARGs and cell number using ARGs-OAP (as Methods 5.3). The results showed that fungal reads had little effect on ARGs-OAP (Table S3).

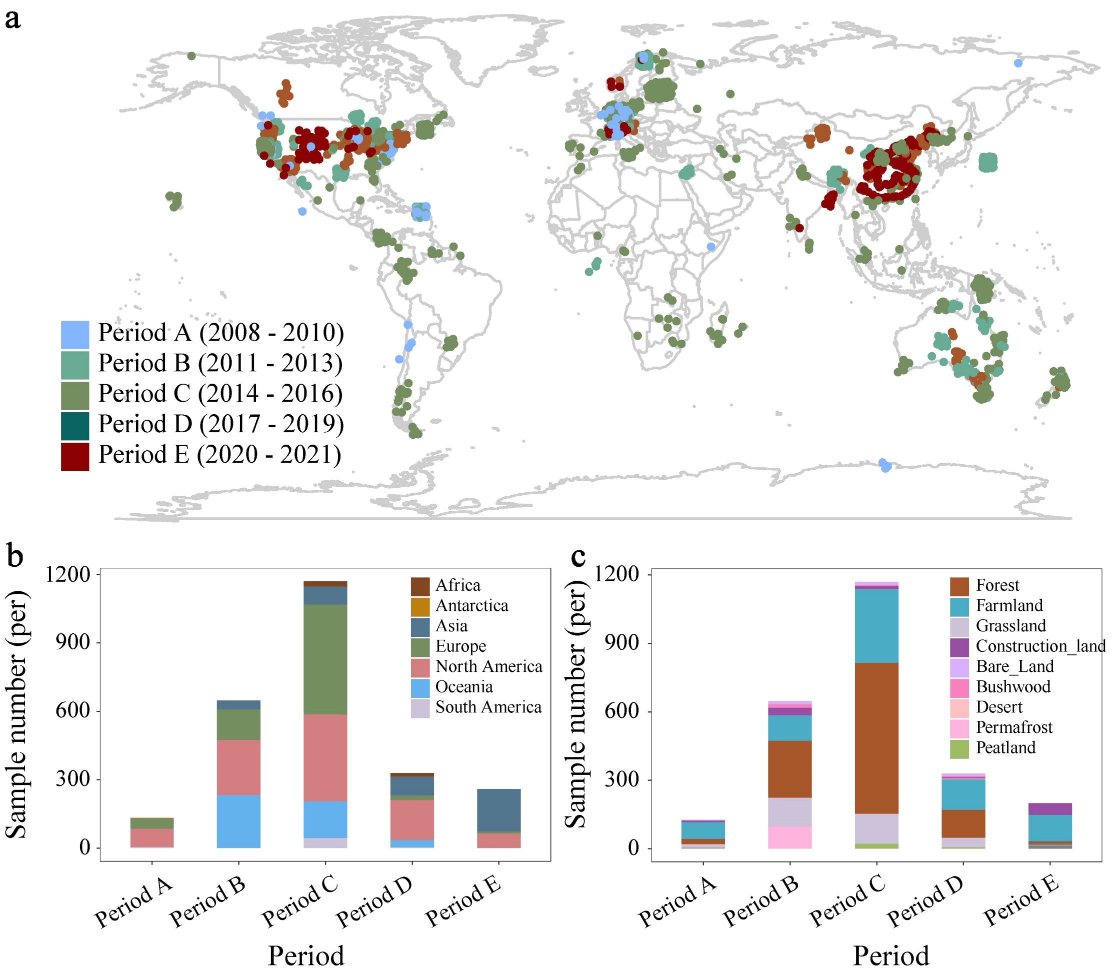

Fig. S1 The global distribution of soil metagenomic data. (a) The global distribution of public and in-house metagenomic data. (b) National information. (c) Habitat information.

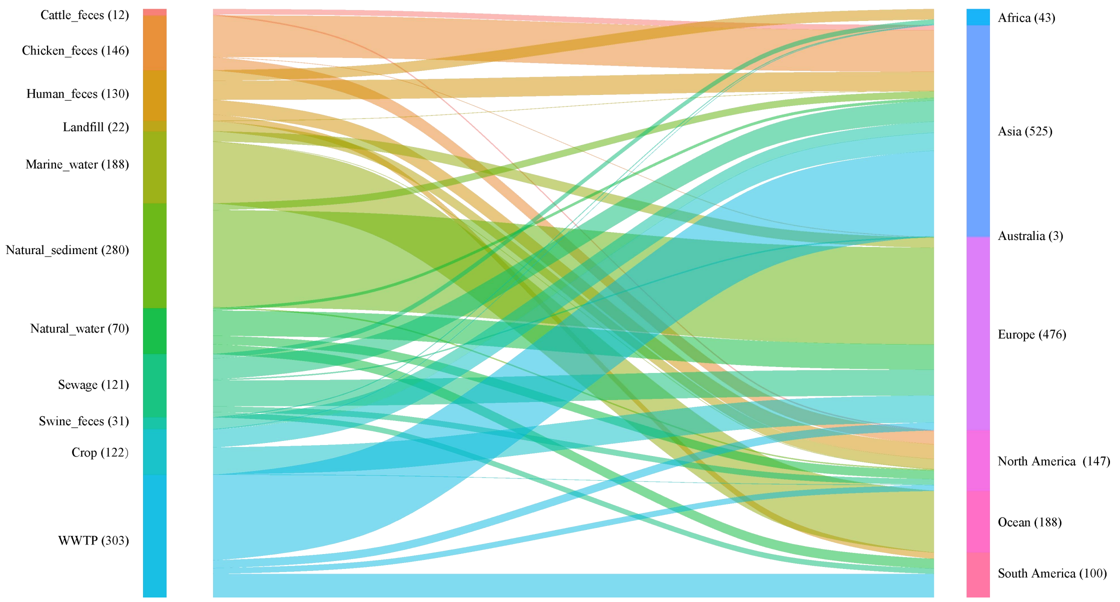

Fig. S2 The distribution of metagenomic data from different habitats.

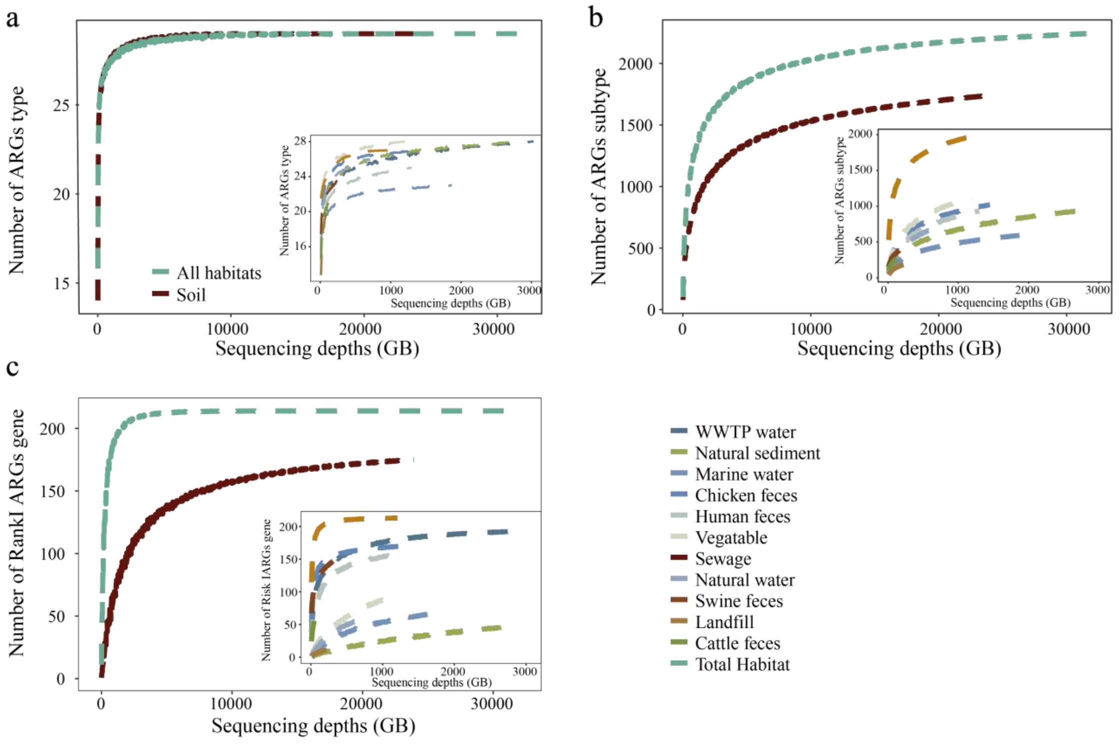

Fig. S3 Rarefaction curves for different habitats. (a) Number of ARG types. The lines in the subplots represent different habitats, while the green line in main plot shows the sum of all habitats. (b) Number of ARG subtypes. The lines in the subplots represent different habitats, while the green line in main plot shows the sum of all habitats. (c) Number of RankI ARG genes. “Total habitat” included all non-soil habitats. The lines in the subplots represent different habitats, while the green line in main plot shows the sum of all habitats.

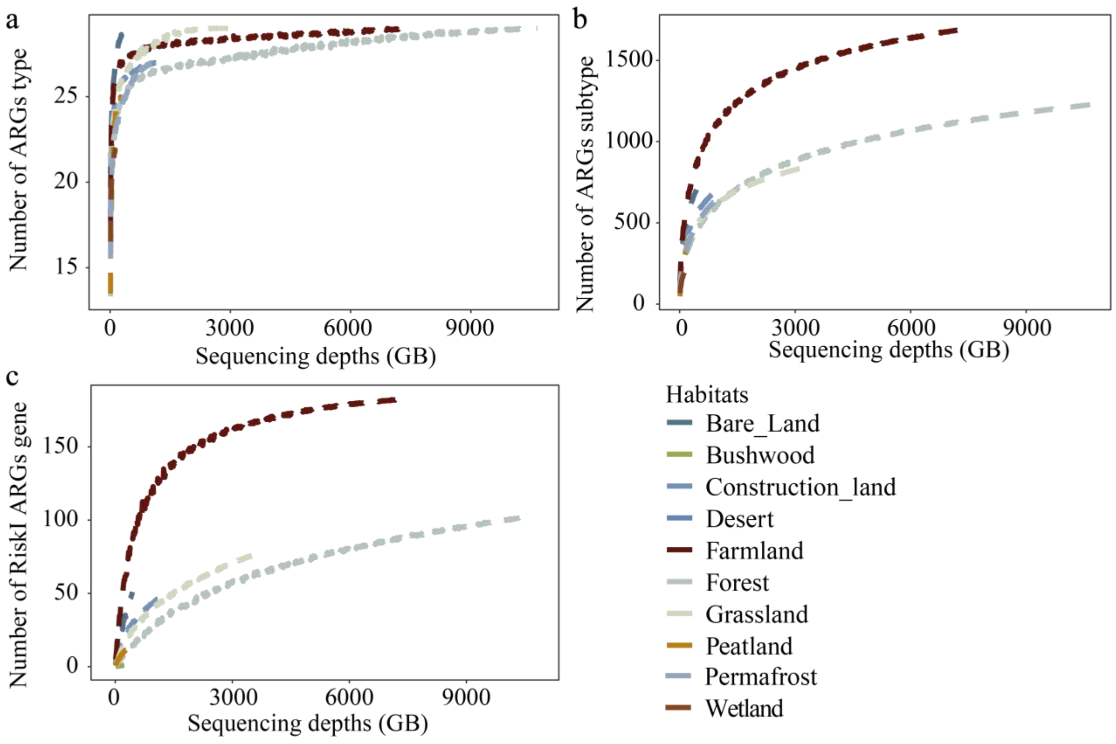

Fig. S4 Soil rarefaction curves grouped by different land use type. (a) Type. (b) Subtype. (c) Gene.

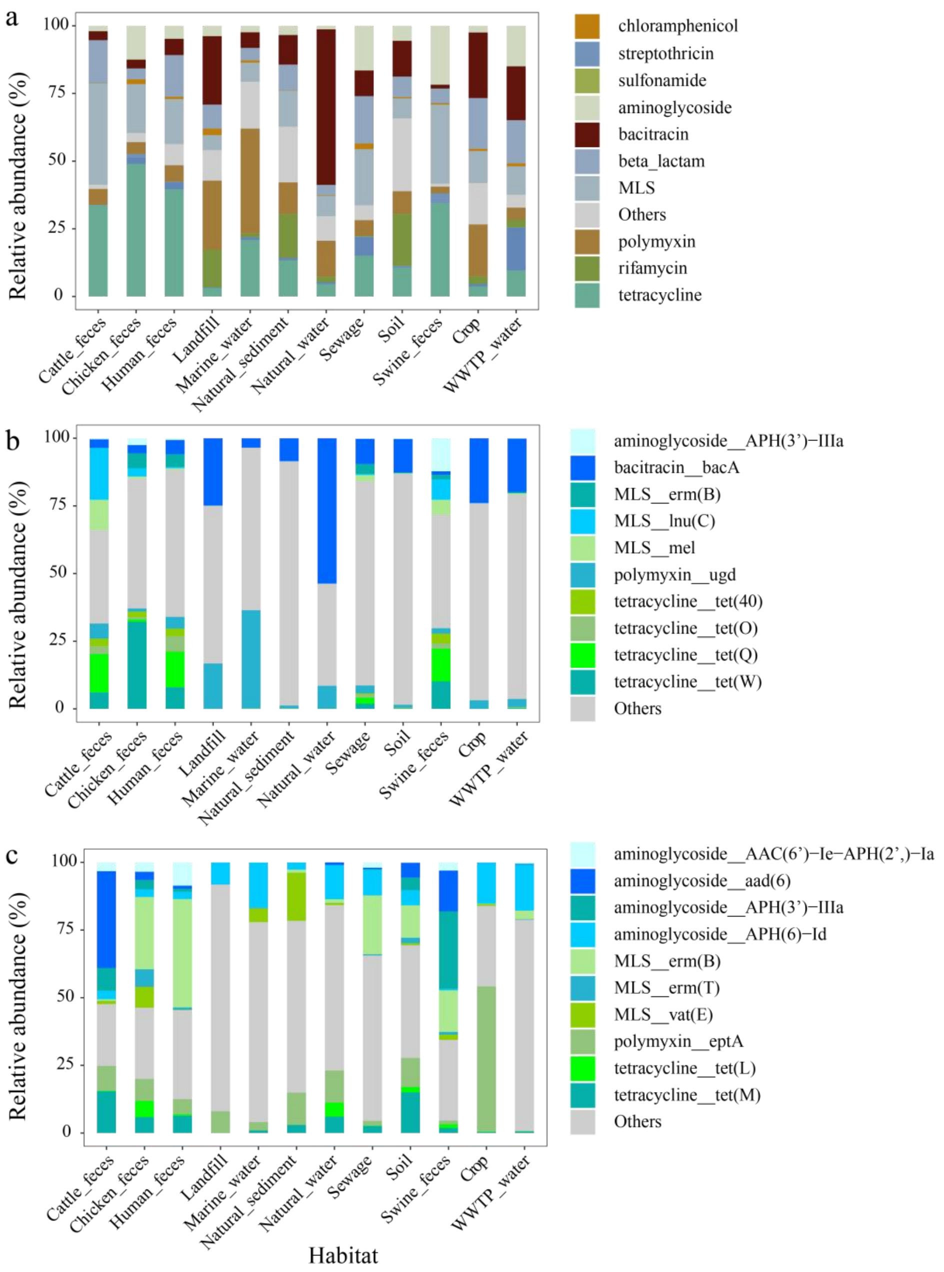

Fig. S5 Composition of Total ARGs and RankI ARGs in different habitats. (a) Composition of Total ARGs in different habitats (type level); (b) Composition of Total ARGs in different habitats (subtype level); (c) Composition of RankI ARGs in different habitats (Subtype level).

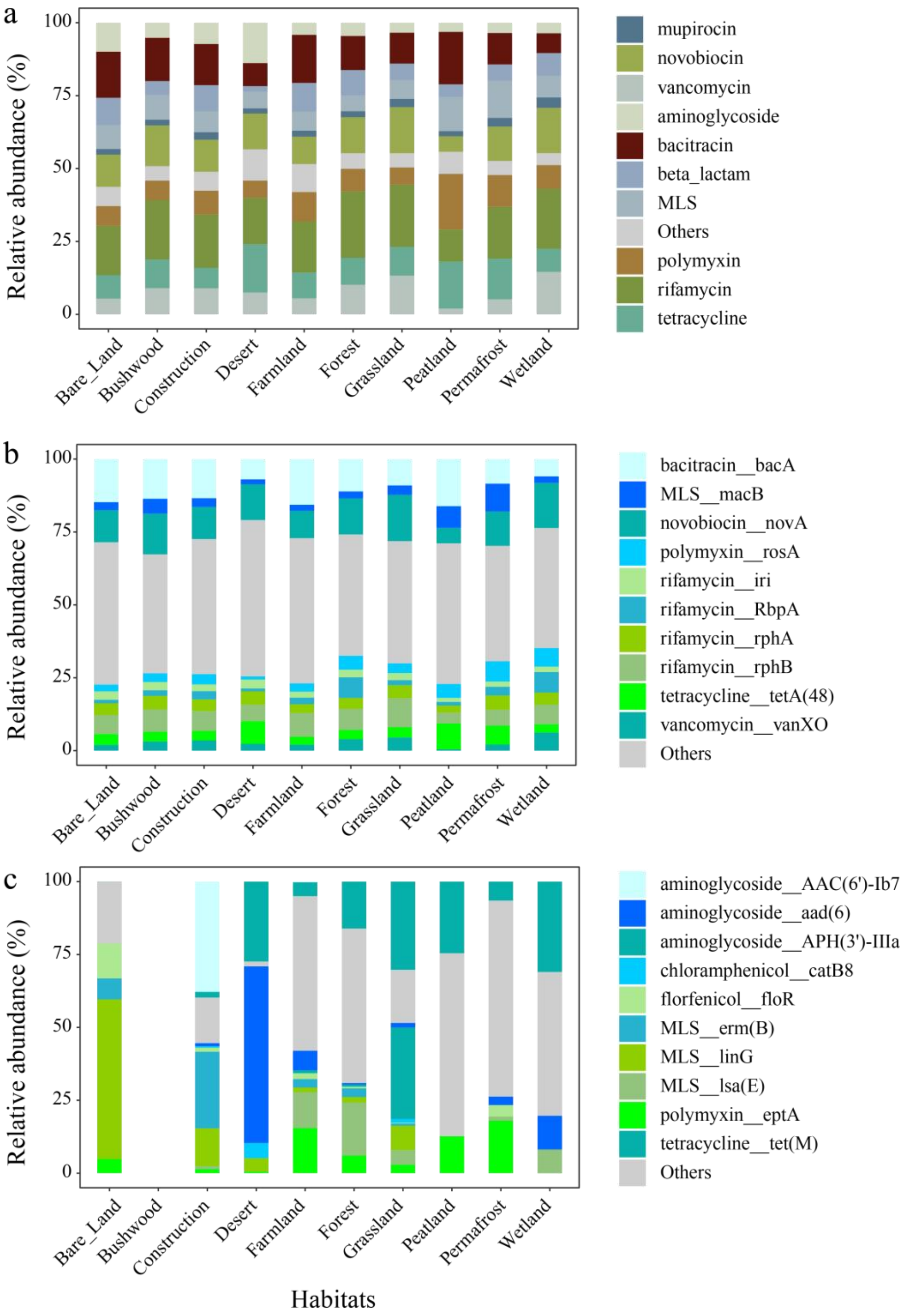

Fig. S6 Composition of Total ARGs and RankI ARGs in different land use type. (a) Composition of Total ARGs in different land use type (type level); (b) Composition of Total ARGs in different land use type (subtype level); (c) Composition of RankI ARGs in different land use type (Subtype level).

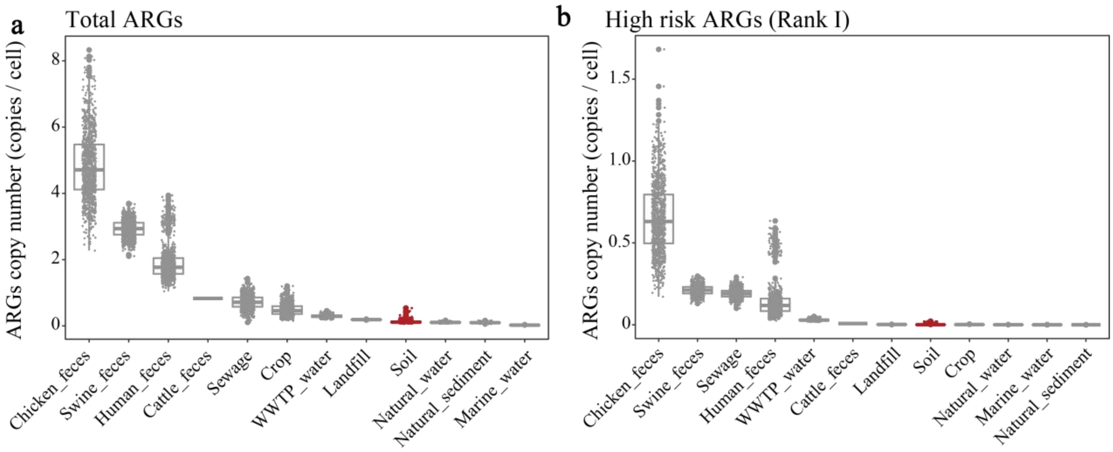

Fig. S7 The relative abundance of Total ARGs and RankI ARGs in different habitats. (a) Total ARGs. In the boxplots of panels, hinges indicate the 25th, 50th, and 75th percentiles, whiskers indicate 1.5× interquartile ranges, dots indicate the average value from each random sampling. Perform 999 rounds of sampling based on the minimum sample size in the habitat (Cattle_feces, 12 samples). (b) RankI ARGs. In the boxplots of panels, hinges indicate the 25th, 50th, and 75th percentiles, whiskers indicate 1.5× interquartile ranges, dots indicate the average value from each random sampling. Perform 999 rounds of sampling based on the minimum sample size in the habitat (Cattle_feces, 12 samples).

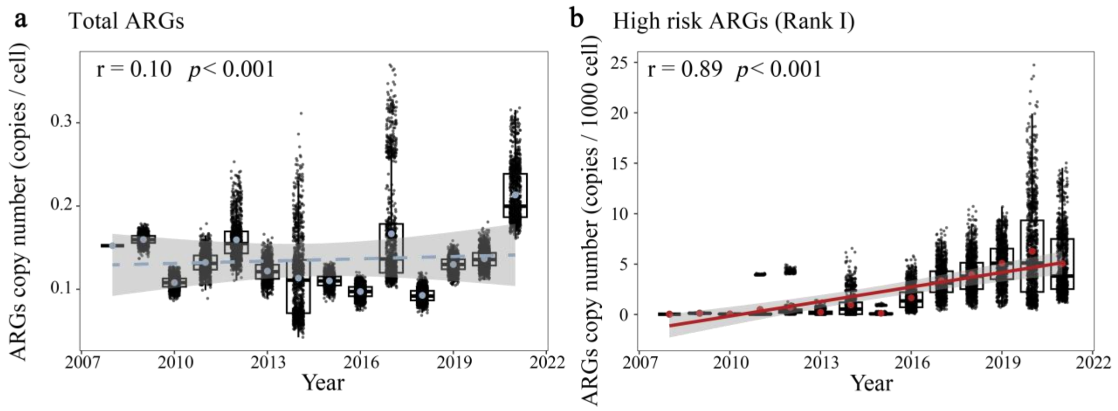

Fig. S8 The relative abundance of Total ARGs and RankI ARGs in different Year. (a) Total ARGs. Perform 999 rounds of sampling based on the minimum sample size in each year (2008, 24 samples). (b) RankI ARGs. Perform 999 rounds of sampling based on the minimum sample size in each year (2008, 24 samples). Pearson correlation test (two-sided) was conducted ($p < 1.5e\text{-}05$).

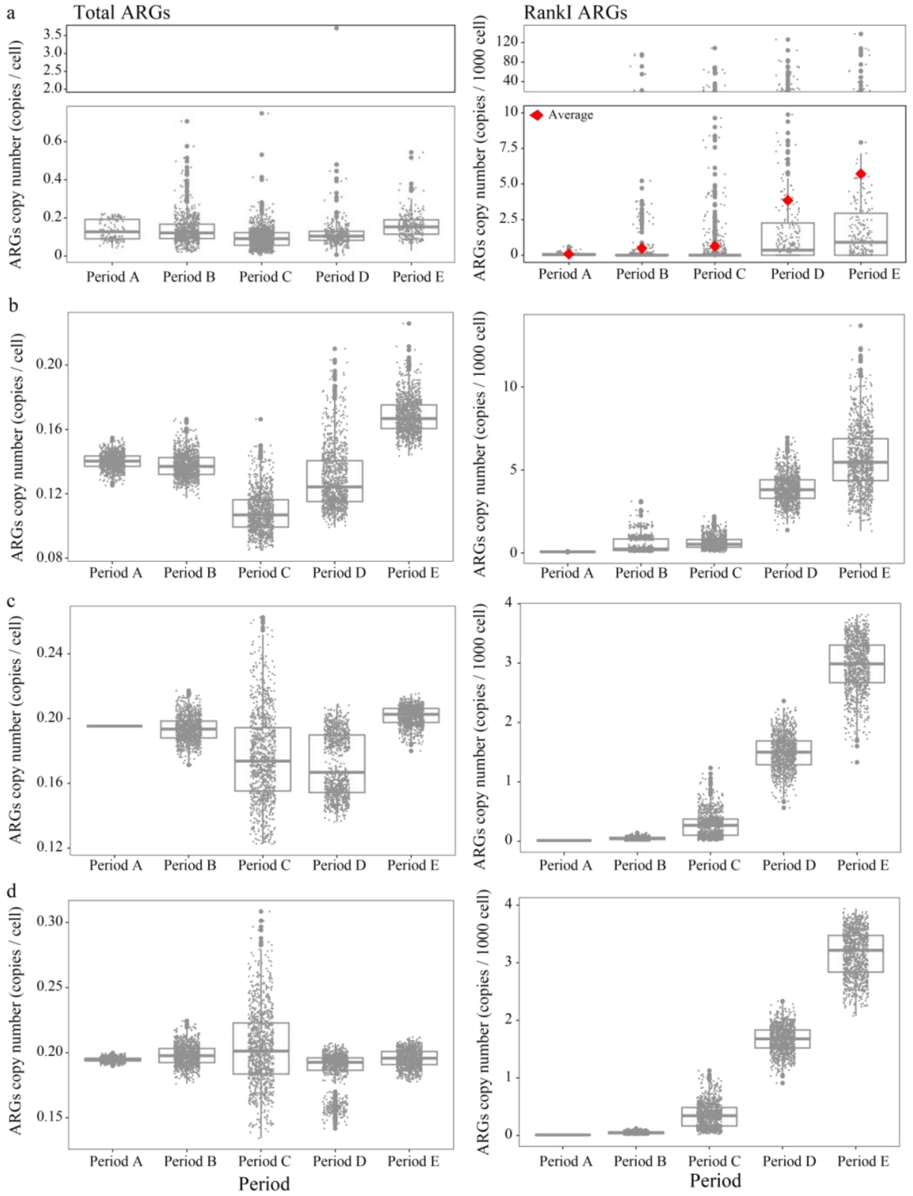

Fig. S9 The relative abundance of Total ARGs and RankI ARGs in different period. (a) Each period uses all of the data. The red dots indicate the means. (b) Perform 999 rounds of sampling based on the minimum sample size in the period (Period A, 133 samples). (c) Perform 999 rounds of sampling based on the specified continent combination (15 from Europe, 15 from America, 10 from Asia, and 10 from others). (d) Perform 999 rounds of sampling based on the specified land use combination (70 from farmland, 20 from forest, 10 from grassland, and 20 from others).

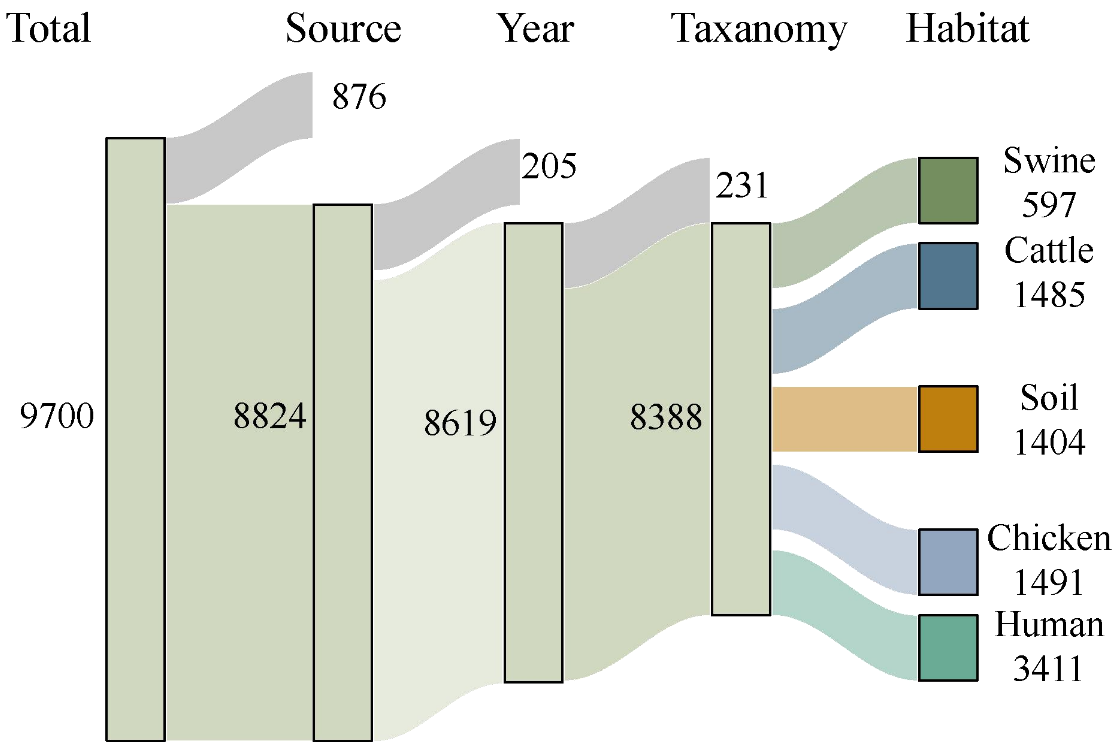

Fig. S10 Principles of screening of *E. coli* isolate genomes

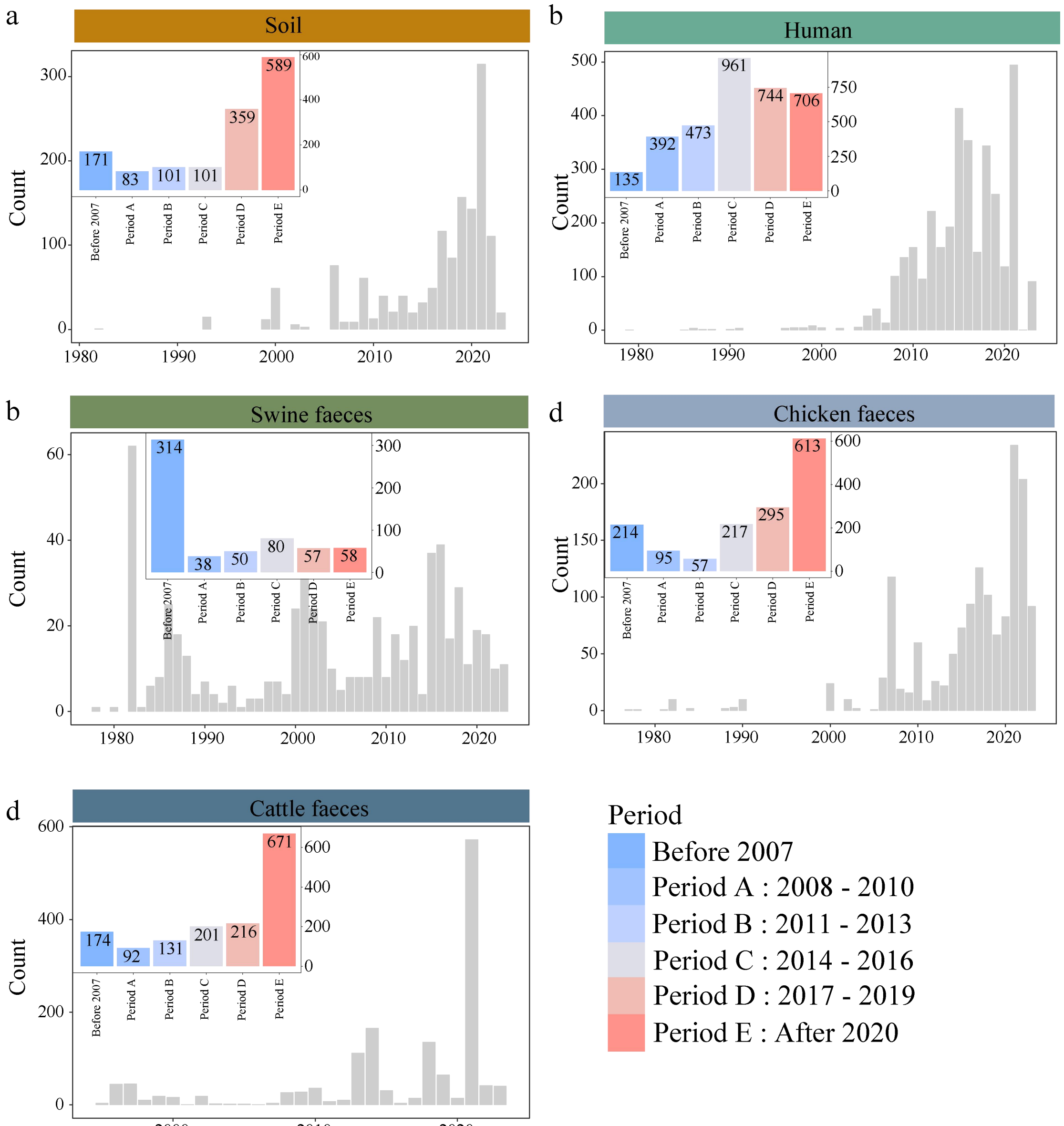

Fig. S11 *E. coli* isolate genomes' number in different period. (a) Soil. (b) Human. (c) Swine faeces. (d) Chicken faeces. (e) Cattle faeces

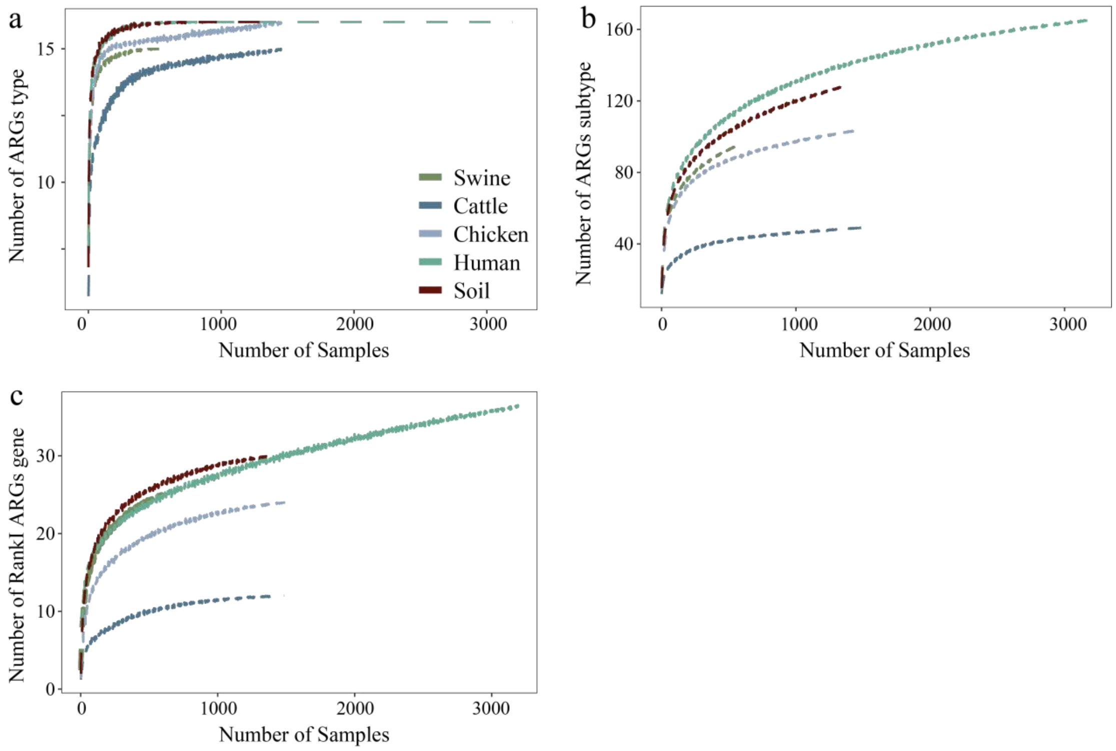

Fig. S12 Rarefaction curves for *E. coli* isolate genomes. (a) Type. (b) Subtype. (c) Type for RankI ARGs.

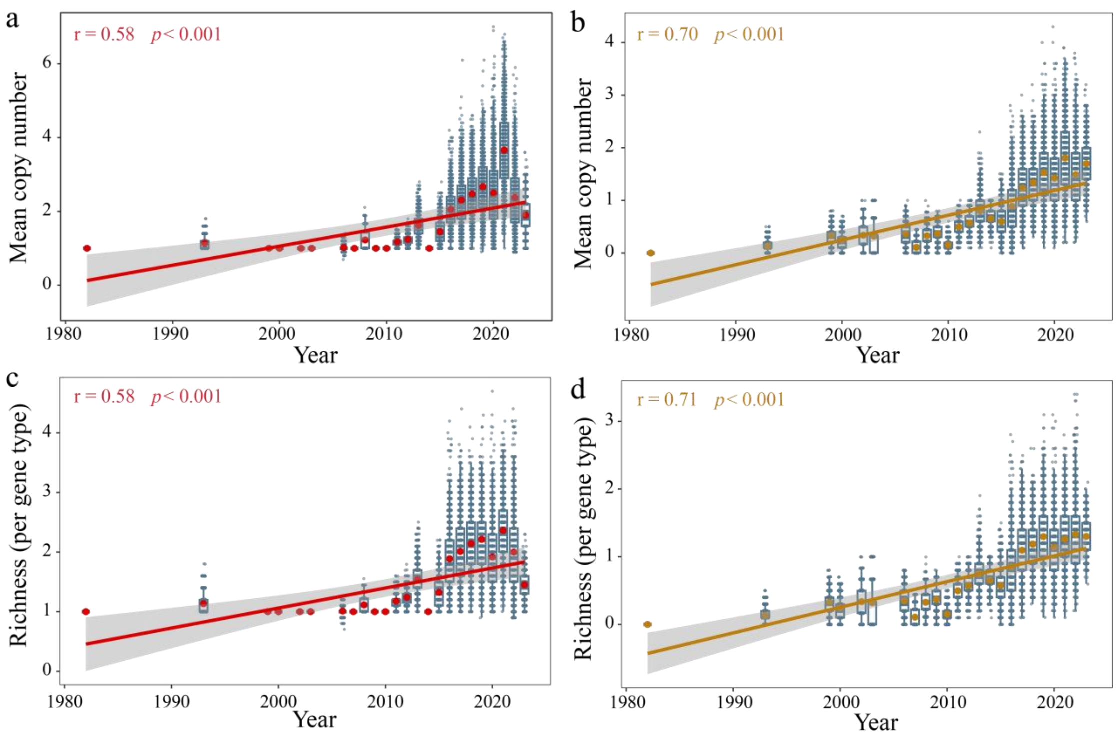

Fig. S13 The relative abundance of RankI ARGs and richness in different Year. (a) Mean copy number of RankI ARGs. Perform 999 rounds of sampling based on the 10 samples in each year. If samples in this year fewer than 10, all will be included in the analysis. Pearson correlation test (two-sided) was conducted (p < 0.00018). (b) Mean copy number of MRankI ARGs. Perform 999 rounds of sampling based on the 10 samples in each year. If samples in this year fewer than 10, all will be included in the analysis. Pearson correlation test (two-sided) was conducted (p < 6.7e-07). (c) Richness of *E. coli* Rank I ARGs (per genome). Perform 999 rounds of sampling based on the 10 samples in each year. If samples in this year fewer than 10, all will be included in the analysis. Pearson correlation test (two-sided) was conducted (p < 0.00015). (d) Richness of *E. coli* MRank I ARGs (per genome). Pearson correlation (loess regression) was conducted to obtained the p value and r. Perform 999 rounds of sampling based on the 10 samples in each year. If samples in this year fewer than 10,

all will be included in the analysis. Pearson correlation test (two-sided) was conducted ($p < 1.3e\text{-}07$).

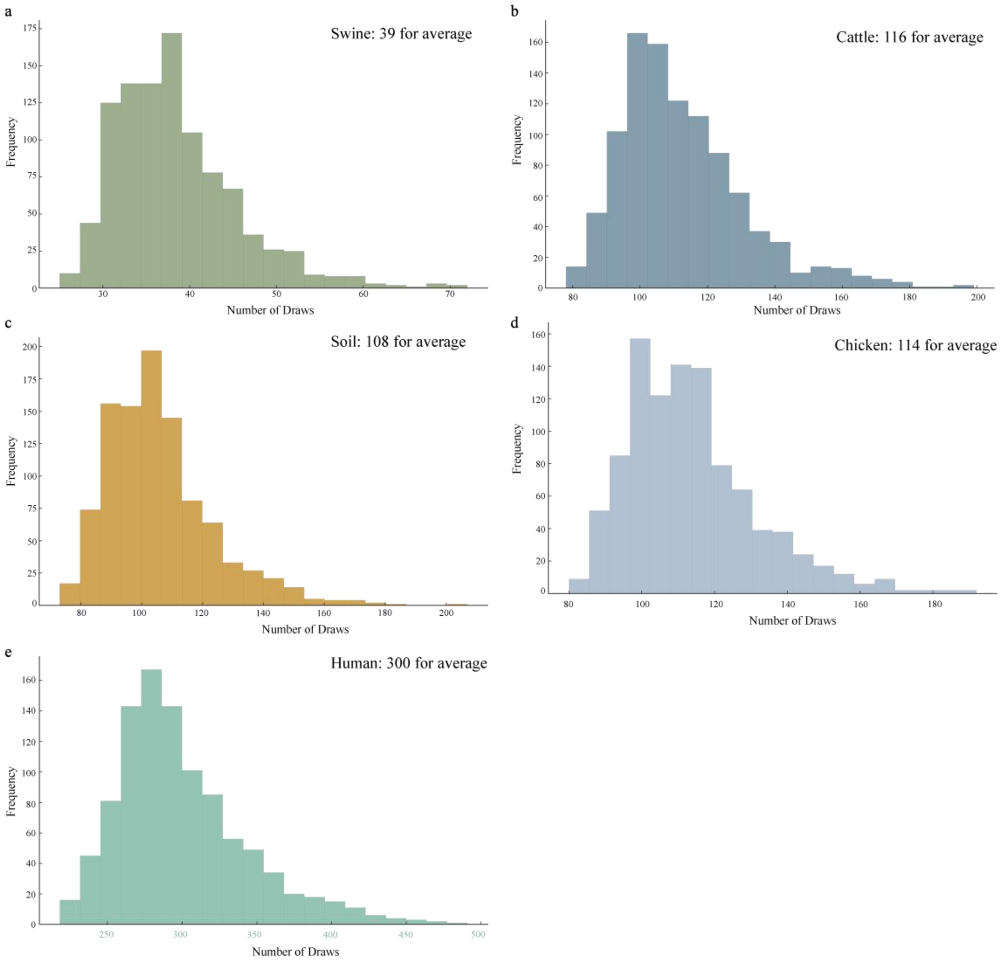

Fig. S14 Number of extractions required to cover all samples. (a) Swine feces. (b) Cattle feces. (c) Soil. (d) Chicken feces. (e) Human.

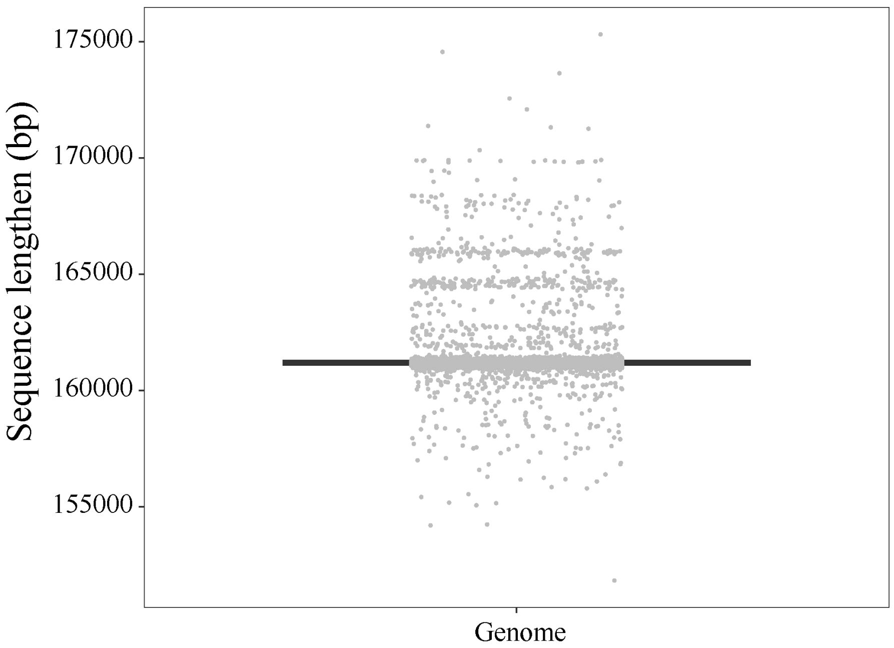

Fig. S15 The length of ribosomal protein (from 8548 genomes).

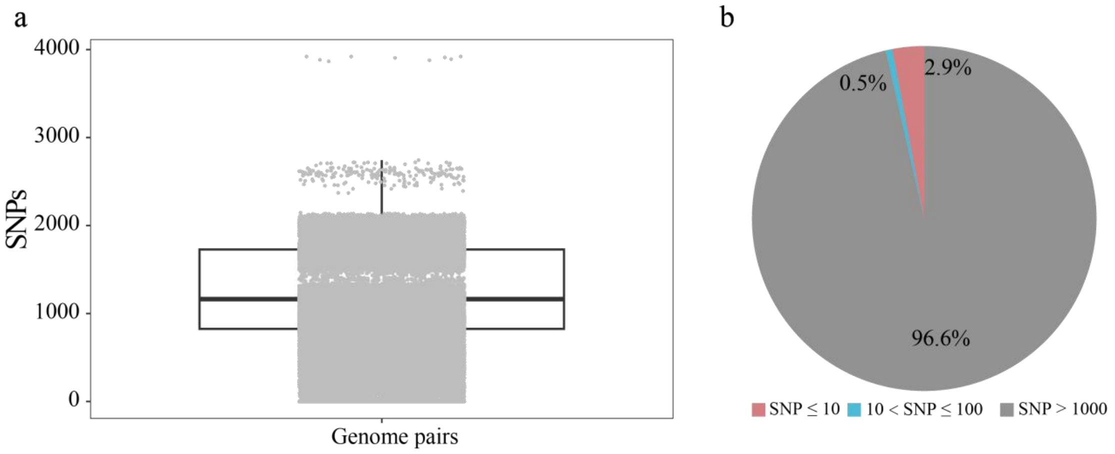

Fig. S16 SNPs for each genome pair. (a) Values of SNPs by different genome pairs (from 8548 genomes). (b) Effect of different cutoff on the number of genome pairs.

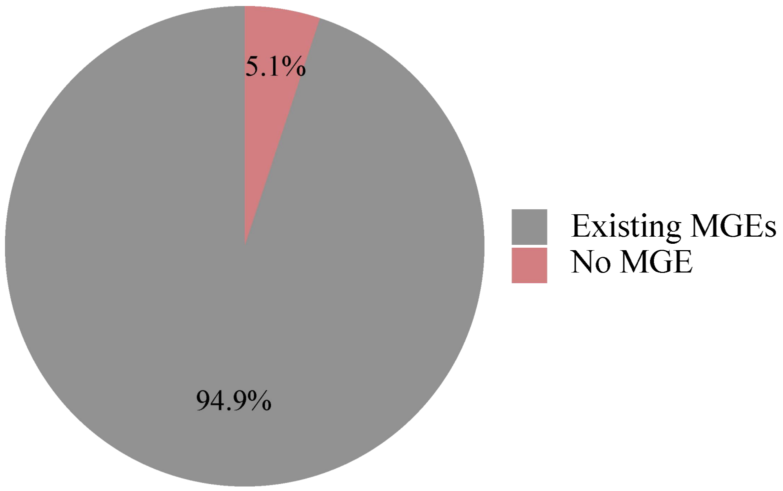

Fig. S17 The detection of MGE for each genome pairs for 500 bp cutoff (present 5 kb upstream and downstream). To confirm the potential HGT, we aligned the RankI ARGs with 5 kb upstream and downstream to Mobile Genetic Element Database *via* diamond blastp (v2.1.8.162)[3].

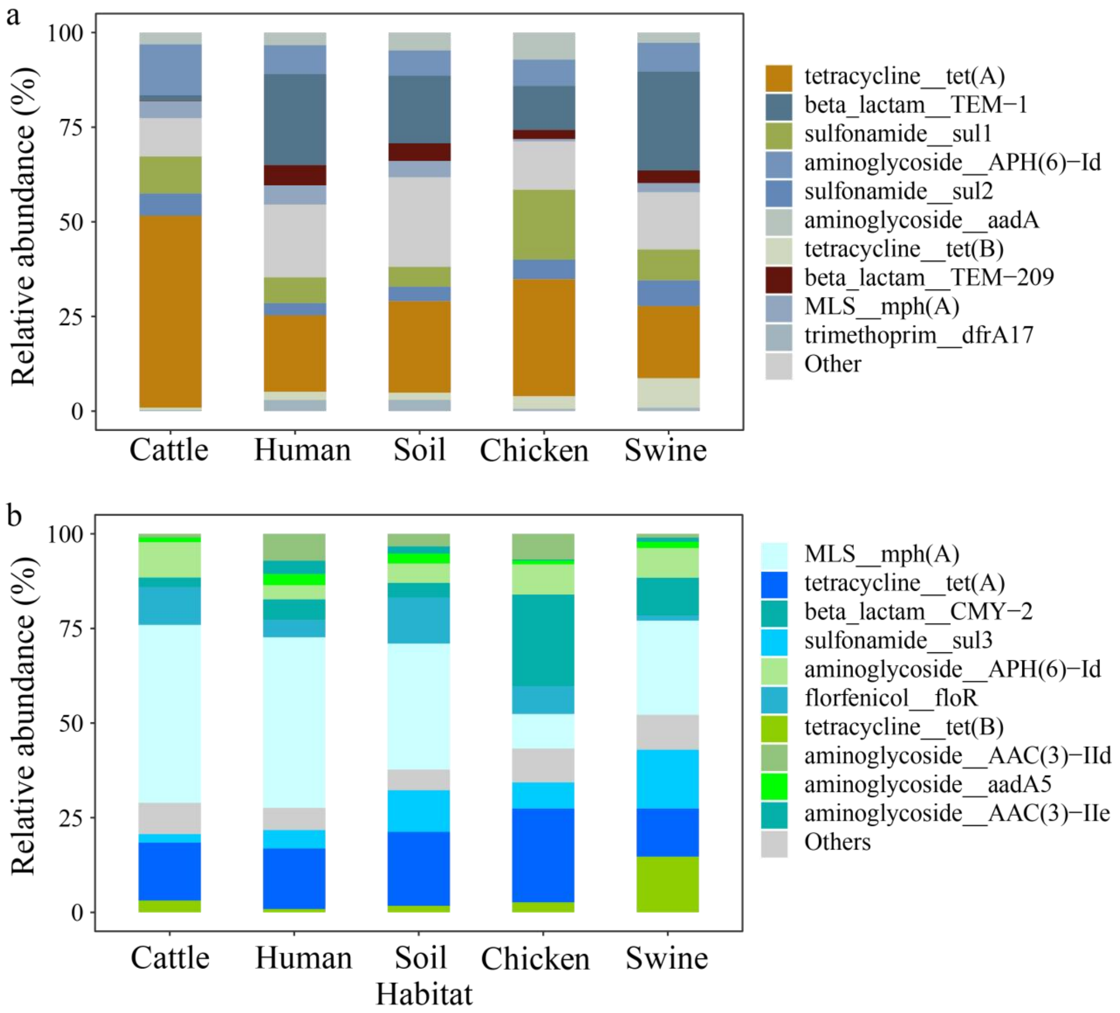

170

171 Fig. S18 Sequence sharing events of total ARGs. (a) Composition of total ARGs

172 including in sequence sharing events. (b) Composition of Rank I ARGs including in

173 sequence sharing events.

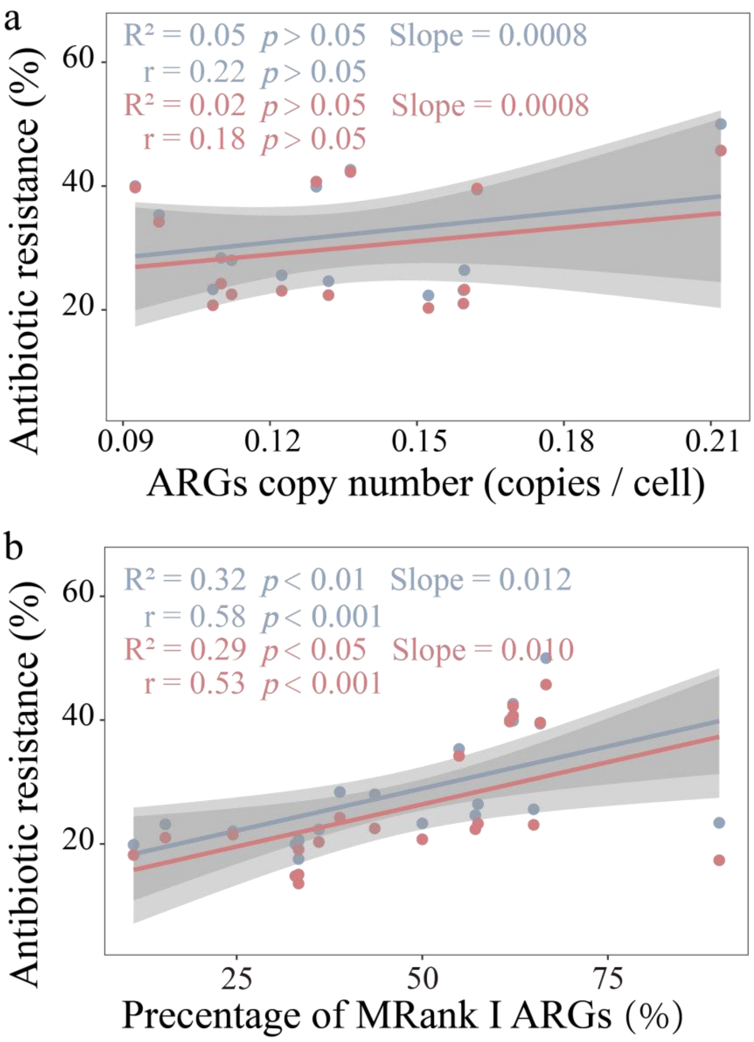

Fig. S19 Relationship between total ARGs in metagenomic samples, mobility RankI ARGs in per E. coli cell and human clinical antibiotic resistance. (a) Total ARGs in metagenomic samples. (b) The relationship between the occurrence frequency of MRank I ARGs in *E. coli* genome (the number of genomes exiting MRank I ARGs in each year / total genome number in that year) and human clinical antibiotic resistance. Grey shading denotes the 95% confidence intervals. The correlation was statistically tested using both Pearson's correlation test (two-sided) and a linear model (two-sided). Total pathogenic bacteria: $p < 4e\text{-}06$, *E.coli*: $p < 2e\text{-}05$.

Tab. S1 Adonis analysis of ARGs composition

Group pairs	Sum.sq	R^2	F	Pr(>F)	Significant
**Total ARGs**	411.5295	0.3057	130.5371	0.001	**Yes**
**RankI ARGs**	0.2583	0.1466	15.9850	0.001	**Yes**

Tab. S2 Multivariate homogeneity of groups dispersions (variances) of ARGs composition

Group pairs	Sum.sq	R^2	F	Pr(>F)	Significant
**Total ARGs**	9.471	0.3238	44.66	0.05	**No**
**RankI ARGs**	18.854	0.2278	215.28	0.05	**No**

Table S3 The influence of fungal reads on ARGs-OAP (v3.2.2)

Sample	Cell for Total dataset	Cell for Fungi dataset	Cell for Fungi / Cell for Total (%)
**Grassland_1**	1333.92	0.21	0.02
**Grassland_2**	1201.18	0.11	0.01
**Forest_1**	1327.46	0.15	0.01
**Forest_2**	1116.07	0.53	0.05
**Forest_3**	1201.81	0.42	0.03
**Forest_4**	3172.60	0.71	0.02
**Forest_5**	1592.62	0.11	0.01
**Forest_6**	1728.15	0.42	0.02
**Farmland_1**	1159.20	0.10	0.01
**Farmland_2**	1445.05	0.33	0.02
**Farmland_3**	1314.12	0.30	0.02
**Construction land**	577.63	0	0.00
**Bare land_1**	304.63	0.05	0.02
**Bare land_2**	404.20	0.02	0.00
**Bare land_3**	281.58	0.03	0.01

References

1. Zhang, A. et al. An omics-based framework for assessing the health risk of antimicrobial resistance genes. *Nat. Commun.* **12**, (2021).
2. Anderson, M. J. A new method for non-parametric multivariate analysis of variance. *Austral Ecol.* **26**, 32-46 (2001).
3. Zhang, Z. et al. Assessment of global health risk of antibiotic resistance genes. *Nat. Commun.* **13**, (2022).
