# Supplementary material for: Global soil antibiotic resistance genes are associated with increasing risk and connectivity to human resistome
Source: Nat Commun. 2025 Aug 4;16:7141. doi: 10.1038/s41467-025-61606-3 (PMC12322111; doi:10.1038/s41467-025-61606-3)
Supplement: Supplementary file 2 — Description of Additional Supplementary Files [file 41467_2025_61606_MOESM2_ESM.pdf]

File Name: Supplementary Data 1

Description: The meta info of all the soil metagenomic samples

File Name: Supplementary Data 2

Description: The meta info of metagenomic samples for other habitats

File Name: Supplementary Data 3

Description: List for Rank I ARGs gene

File Name: Supplementary Data 4

Description: Pairwise Adonis analysis of Total ARGs

File Name: Supplementary Data 5

Description: Pairwise Adonis analysis of Rank I ARGs

File Name: Supplementary Data 6

Description: Representative ARGs (type subtype and gene)

File Name: Supplementary Data 7

Description: List for prokaryotic pathogens

File Name: Supplementary Data 8

Description: The info of the isolation E.coli genome

File Name: Supplementary Data 9

Description: Detailed *p* value and T stat for the copy number for Rank I ARGs and MRank I ARGs in *E.coli* genome

File Name: Supplementary Data 10

Description: Detailed *p* value and T stat for the copy number for the richness of Rank I ARGs and MRankI ARGs in *E.coli* genome

File Name: Supplementary Data 11

Description: Detailed *p* value and T stat for the copy number for the FEAST analysis

File Name: Supplementary Data 12

Description: Detailed *p* value and T stat for the copy number for the connectedness (*eptA*)

File Name: Supplementary Data 13

Description: Detailed *p* value and T stat for the copy number for the sequence sharing events with SNPs over 1000
