# Supplementary material for: Global soil antibiotic resistance genes are associated with increasing risk and connectivity to human resistome
Source: Nat Commun. 2025 Aug 4;16:7141. doi: 10.1038/s41467-025-61606-3 (PMC12322111; doi:10.1038/s41467-025-61606-3)
Supplement: Supplementary file 4 — Reporting Summary [file 41467_2025_61606_MOESM4_ESM.pdf]

nature portfolio

Corresponding author(s): Tong Zhang

Last updated by author(s): May 15, 2025

Reporting Summary

Nature Portfolio wishes to improve the reproducibility of the work that we publish. This form provides structure for consistency and transparency in reporting. For further information on Nature Portfolio policies, see our Editorial Policies and the Editorial Policy Checklist.

Statistics

For all statistical analyses, confirm that the following items are present in the figure legend, table legend, main text, or Methods section.

n/a	Confirmed	
☐	☒	The exact sample size (*n*) for each experimental group/condition, given as a discrete number and unit of measurement
☐	☒	A statement on whether measurements were taken from distinct samples or whether the same sample was measured repeatedly
☐	☒	The statistical test(s) used AND whether they are one- or two-sided<br>*Only common tests should be described solely by name; describe more complex techniques in the Methods section.*
☐	☒	A description of all covariates tested
☐	☒	A description of any assumptions or corrections, such as tests of normality and adjustment for multiple comparisons
☐	☒	A full description of the statistical parameters including central tendency (e.g. means) or other basic estimates (e.g. regression coefficient) AND variation (e.g. standard deviation) or associated estimates of uncertainty (e.g. confidence intervals)
☐	☒	For null hypothesis testing, the test statistic (e.g. *F*, *t*, *r*) with confidence intervals, effect sizes, degrees of freedom and *P* value noted<br>*Give P values as exact values whenever suitable.*
☒	☐	For Bayesian analysis, information on the choice of priors and Markov chain Monte Carlo settings
☒	☐	For hierarchical and complex designs, identification of the appropriate level for tests and full reporting of outcomes
☐	☒	Estimates of effect sizes (e.g. Cohen's *d*, Pearson's *r*), indicating how they were calculated

Our web collection on statistics for biologists contains articles on many of the points above.

Software and code

Policy information about availability of computer code

Data collection	The public data were downloaded using Aspera v 4.2.12.780 (https://www.ibm.com/cn-zh/products/aspera).
Data analysis	Both public metagenomic data (11 habitats) and in-house metagenomic data were analyzed using the same framework. In detail, Trimmomatic (v.0.36) was used to filter out low quality reads (leading: 3, trailing:3, slidingwindow:4:20, minlen:100). ARG profiles were quantified using ARGs-OAP (v3.2.2) with a cut-off of 80% identity, 75% coverage, and 1e-7 e-value. The clean data for 2,540 soil metagenomic datasets (i.e., 149 in-house data and 2,391 public data) were assembled by MEGAHIT (v1.2.9, parameters: k-min 35, k-max 115, k-step 20) to obtain contigs61. After filtering contigs less than 5,000 bp, prodigal (v2.6.3) was used to predict the ORFs of contigs. To identify the ARG-carrying contigs, the ORFs were aligned to the SARG (v3.2.1-S)26 via diamond blastp (v2.1.8.162). The ARG-carrying contigs were further annotated by kraken2 base on the GTDB (Release 220). Bowtie2 (version 2.4.2) was used for sequence alignment (very-sensitive), and Samtools (version 1.11) was used to process and convert the alignment results. The taxonomy and phylogeny for E.coli isolate genomes were further checked using the Genome Taxonomy Database Toolkit (GTDB-Tk; v2.4.0), resulting in the exclusion of 231 genomes. Prodigal (v2.6.3) was used to predict the ORFs of each E. coli genome. To obtain the profile of ARGs and MGEs, the ORFs of these genomes were aligned to the SARG (v3.2.1-S) and Mobile Genetic Element Database via diamond blastp (v2.1.8.162). The sequences were aligned using clustwal (v2.1) and phylogenetic trees were constructed with FastTree (v2.1). Blastn (v2.6.0) was used to screen blocks of DNA that were shared by two genomes.

For manuscripts utilizing custom algorithms or software that are central to the research but not yet described in published literature, software must be made available to editors and reviewers. We strongly encourage code deposition in a community repository (e.g. GitHub). See the Nature Portfolio guidelines for submitting code & software for further information.

Data

Policy information about availability of data

All manuscripts must include a data availability statement. This statement should provide the following information, where applicable:

- Accession codes, unique identifiers, or web links for publicly available datasets
- A description of any restrictions on data availability
- For clinical datasets or third party data, please ensure that the statement adheres to our policy

The in-house metagenomic sequencing data generated in this study have been deposited in the National Center for Biotechnology Information (NCBI) Sequence Read Archive (SRA) database under accession number PRJNA1202346 [https://www.ncbi.nlm.nih.gov/bioproject/PRJNA1202346] and PRJNA1229199 [https://www.ncbi.nlm.nih.gov/bioproject/PRJNA1229199]. All online metagenomic sequencing data and E. coli isolate genomes used in this study are available in the NCBI RefSeq database, IMG/M portal, and European Nucleotide Archive. Information for all metadata used in this study as well as the important data for analysis are provided in Supplementary Data 1, 2, and 8. Source data are provided as a Source Data file.

Research involving human participants, their data, or biological material

Policy information about studies with human participants or human data. See also policy information about sex, gender (identity/presentation), and sexual orientation and race, ethnicity and racism.

Reporting on sex and gender	This information has not been collected.
Reporting on race, ethnicity, or other socially relevant groupings	This information has not been collected.
Population characteristics	See above.
Recruitment	See above.
Ethics oversight	See above.

Note that full information on the approval of the study protocol must also be provided in the manuscript.

Field-specific reporting

Please select the one below that is the best fit for your research. If you are not sure, read the appropriate sections before making your selection.

☐ Life sciences ☐ Behavioural & social sciences ☒ Ecological, evolutionary & environmental sciences

For a reference copy of the document with all sections, see nature.com/documents/nr-reporting-summary-flat.pdf

Ecological, evolutionary & environmental sciences study design

All studies must disclose on these points even when the disclosure is negative.

Study description	We analyzed 3,816 metagenomic data (including 11 habitats) and 8,388 genomes of Escherichia coli isolates (the main pathogen indicator in soil) in the public databases together with some in-house metagenomic samples (149 soil samples). Metagenomic and E. coli genome analysis provided evidence that the risk of ARGs in soil, measured by Rank I ARGs, has increased over time. We introduced a novel "connectedness" metric, a phylogeny-based approach to assess the association of ARGs across different habitats and revealed that the soil antibiotic resistome was increasingly related to the human antibiotic resistome. The comparison of 45 million genome pairs also supported this finding and suggested that cross-habitat horizontal gene transfer (HGT) played a crucial role in the connectivity of ARGs between humans and soil. Moreover, we compiled datasets on human clinical antibiotic resistance (covering 126 countries from 1998 to 2022) and found significant correlations between the relative abundance (metagenomic), copy number (isolation genome) of Rank I ARGs and the proportion of potential HGT events and human clinical antibiotic resistance ($R2 = 0.41 - 0.82$, $p < 0.001$). Overall, our work provided insights into the relationship and transmission pathways of ARGs between soil and humans, and could help identify control strategies against antibiotic resistance and its dissemination.
Research sample	We included four part of dataset in our manuscript, including soil metagenomic dataset, various habitats metagenomic dataset, Genomes of E.coli isolate in various habitats, and human clinical antibiotic resistance dataset.<br>(1)Soil metagenomic dataset: The soil dataset contained 2,540 samples, of which 2,391 public samples were public data and 149 were in-house data.<br>(2)Various habitats metagenomic dataset：The various habitats dataset contained 1,425 public samples, collected from different habitats, such as swine faeces, cattle faeces, chicken faeces, human faeces, water from wastewater treatment plants, sewage, natural sediment, natural water, marine water, crop surface and landfill.<br>(3)Genomes of E.coli isolate in various habitats: 8,388 E. coli public isolate genomes were used, including 1404 from soil, 1485 from cattle faeces, 1491 from chicken faeces, 3411 from humans, and 597 from swine faeces.<br>(4)Human clinical antibiotic resistance dataset: The dataset contained data collected from 126 countries between 1998 and 2022.
Sampling strategy	A total of 149 Chinese soil samples were collected in 2018 (36 samples) and 2021 (113 samples). Briefly, the upper soil layer was

	collected using a soil auger and each sample was composed of five sub-samples (i.e., 4 corners and the center) to eliminate heterogeneity. After removing plant roots and stones from the soil, the fresh soil was stored at -20℃.
Data collection	(1)The public raw data for soil metagenomic dataset, various habitats metagenomic dataset and genomes of E.coli isolate in various habitats were downloaded using Aspera (https://www.ibm.com/cn-zh/products/aspera) from NCBI.<br>(2)We collected the human clinical antibiotic resistance genes from 5 sources, including Resistancemap, European Centre for Disease Prevention and Control Surveillance Atlas, PLISA Health Information Platform for the Americas, World Health Organization, and China Antimicrobial Resistance Monitoring System. The dataset included 126 countries, 18 pathogens, 53 antibiotic agents, and was collected from 1998-2022. The definition of antibiotic resistance was that the tested strains were non-susceptible (i.e., intermediate or resistant) to an antibiotic.
Timing and spatial scale	In house Chinese soil samples were collected in 2018 (36 samples) and 2021 (113 samples). The public soil metagenomic dataset, various habitats metagenomic dataset, genomes of E.coli isolate in various habitats and human clinical antibiotic resistance dataset were collected and downloaded in 2023.
Data exclusions	(1) Metagenomic dataset (including soil and other habitats): To minimize possible bias, we only included public data that fulfilled the following criteria (i) Illumina shotgun data; (ii) paired-end data with FASTQ format; (iii) over 1GB; (iv); (v) no culturing or any other additional experiments; (vi) not collected from potentially contaminated environments; (vii) included detailed sample information (e.g., accurate coordinate and sampling time); (viii) average read length over 100 base pairs. These criteria represented an initial quality control of the soil dataset to minimise the influence of various sources on the results.<br>(2) Genomes of E.coli isolate in various habitats: As E. coli is one of the most important prokaryotic pathogens in soil, we downloaded the genomes of 9700 E. coli isolates from NCBI. We only included the genomes of E. coli isolates that fulfilled the following criteria: (i) isolated from soil, human, chicken faeces, cattle faeces, and swine faeces; (ii) clear isolation information, including isolation country and isolation date; (iii) similar genome number of different isolation sources. The taxonomy and phylogeny were further checked using the Genome Taxonomy Database Toolkit (GTDB-Tk; v2.4.0), resulting in the exclusion of 231 genomes. In total, 8,388 E. coli isolate genomes were used, including 1404 from soil, 1485 from cattle faeces, 1491 from chicken faeces, 3411 from humans, and 597 from swine faeces(Supplementary Data 7). These E. coli were isolated between 1977 and 2023 from 53 countries. The completeness of all genomes was > 97%, and the contamination level was < 5% (Supplementary Data 7).<br>(3) Human clinical antibiotic resistance dataset: The definition of antibiotic resistance was that the tested strains were non-susceptible (i.e., intermediate or resistant) to an antibiotic. To eliminate the effect of sample size, isolation rates (antibiotic resistance bacteria / total tested bacteria) were focused on and only bacteria with a total number greater than 30 were included.
Reproducibility	The datasets we used were validated through rarefaction curves, indicating that the data represent the composition of ARGs, with minimal impact on the results even if additional samples were included. In terms of statistical analysis, we validated the same results across different datasets, including metagenomes, E.coli isolate genomes, and human clinical antibiotic resistance dataset. For temporal trends, we resampled the data, including the original dataset, a dataset normalized to the same sample size, a dataset rarefied to the same depth based on the minimum sample size in the earliest time period, and a dataset rarefied to the same depth by fixing the sample size across continents and the minimum sample size in the earliest time period.
Randomization	We divided the data into five time periods, including Period A: 2008 - 2010, Period B: 2011 - 2013, Period C: 2014 - 2016, Period D: 2017 - 2019, Period E: 2020 - 2021.
Blinding	There is no need for blinding in the our analysis.

Did the study involve field work? ☐ Yes ☒ No

Reporting for specific materials, systems and methods

We require information from authors about some types of materials, experimental systems and methods used in many studies. Here, indicate whether each material, system or method listed is relevant to your study. If you are not sure if a list item applies to your research, read the appropriate section before selecting a response.

Materials & experimental systems

n/a	Involved in the study
☒	☐ Antibodies
☒	☐ Eukaryotic cell lines
☒	☐ Palaeontology and archaeology
☒	☐ Animals and other organisms
☒	☐ Clinical data
☒	☐ Dual use research of concern
☒	☐ Plants

Methods

n/a	Involved in the study
☒	☐ ChIP-seq
☒	☐ Flow cytometry
☒	☐ MRI-based neuroimaging

Plants

Seed stocks

Report on the source of all seed stocks or other plant material used. If applicable, state the seed stock centre and catalogue number. If plant specimens were collected from the field, describe the collection location, date and sampling procedures.

Novel plant genotypes

Describe the methods by which all novel plant genotypes were produced. This includes those generated by transgenic approaches, gene editing, chemical/radiation-based mutagenesis and hybridization. For transgenic lines, describe the transformation method, the number of independent lines analyzed and the generation upon which experiments were performed. For gene-edited lines, describe the editor used, the endogenous sequence targeted for editing, the targeting guide RNA sequence (if applicable) and how the editor was applied.

Authentication

Describe any authentication procedures for each seed stock used or novel genotype generated. Describe any experiments used to assess the effect of a mutation and, where applicable, how potential secondary effects (e.g. second site T-DNA insertions, mosiacism, off-target gene editing) were examined.
